# Supplementary material for: Metastable spiking networks in the replica-mean-field limit
Source: PLoS Comput Biol. 2022 Jun 17;18(6):e1010215. doi: 10.1371/journal.pcbi.1010215 (PMC9246178; doi:10.1371/journal.pcbi.1010215)
Supplement: S1 Appendix — A: Derivation of the DDEs, B: Simulation method, C: Calculation for higher moments, D: Different approximation methods, E: Prediction of dynamical transition rates. Fig A: Bistable networks. Fig B: State switching paths. (PDF) [file pcbi.1010215.s001.pdf]

S1 Appendix: Supplementary text

Luyan Yu[1], Thibaud Taillefumier[2, 3*]

1 Department of Physics, University of Texas at Austin, Austin, Texas, USA
2 Department of Mathematics, University of Texas at Austin, Austin, Texas, USA
3 Department of Neuroscience, University of Texas at Austin, Austin, Texas, USA

* ttaillef@austin.utexas.edu

A Derivation of the DDEs

In the main text, we claim the MGF L_i satisfies the DDE. We derive it here from the rate-conservation principle on the MGF, which we recall here:

$$\begin{aligned} e^{ux_i(t)} - e^{ux_i(0)} = & -\frac{u}{\tau_i}\int_0^t x_i(s)e^{ux_i(s)}\,\mathrm{d}s \\ & + \sum_{j\neq i}\left(e^{u\mu_{ji}}-1\right)\int_0^t e^{ux_i(s^-)}P_j(\mathrm{d}s) \\ & + \int_0^t \left(1-e^{ux_i(s^-)}\right)N_i(\mathrm{d}s)\,. \end{aligned} \tag{1}$$

First, we take the expectation value on both sides and the L.H.S. becomes zero in stationary limit:

$$\begin{aligned} 0 = & -\frac{u}{\tau_i}\,\mathbb{E}\left[\int_0^t x_i(s)e^{ux_i(s)}\,\mathrm{d}s\right] \\ & + \sum_{j\neq i}\left(e^{u\mu_{ji}}-1\right)\mathbb{E}\left[\int_0^t e^{ux_i(s^-)}P_j(\mathrm{d}s)\right] \\ & + \ \mathbb{E}\left[\int_0^t \left(1-e^{ux_i(s^-)}\right)N_i(\mathrm{d}s)\right]. \end{aligned} \tag{2}$$

The first term is an ordinary integral and in stationary limit, it becomes

$$\begin{aligned} -\frac{u}{\tau_i}\,\mathbb{E}\left[\int_0^t x_i(s)e^{ux_i(s)}\,\mathrm{d}s\right] & = -\frac{u}{\tau_i}t\,\mathbb{E}\left[x_i e^{ux_i}\right] \\ & = -\frac{u}{\tau_i}tL_i'(u). \end{aligned} \tag{3}$$

The stochastic integrals in the second and third terms involving point processes can be handled using Palm calculus [1]. Palm calculus is established on Palm probability measure, which intuitively is the probability viewed from the event occurrence time of given point process. Here we state the key result from Palm calculus. For stationary process $X(t)$ with some mild conditions, the expectation of the integral on point process $N(t)$ of rate β has the following relation:

$$\mathbb{E}\left[\int_0^t X(s)N(\mathrm{d}s)\right] = \beta t\,\mathbb{E}_N^0\left[X(0^-)\right], \tag{4}$$

where $\mathbb{E}_N^0[\cdot]$ denotes the expectation with respect to Palm probability. Further, if there is a stochastic intensity process $\lambda(t)$ associated with the point process $N(t)$, we have for arbitrary real-valued function f,

$$\beta\,\mathbb{E}_N^0\left[f[X(0^-)]\right] = \mathbb{E}\left[f(X)\lambda\right]. \tag{5}$$

For the expectation in the sum of the second term, we use Eq. (4) and it becomes

$$\begin{aligned}\mathbb{E}\left[\int_0^t e^{ux_i(s^-)} P_j(\mathrm{d}s)\right] &= \beta_j t\,\mathbb{E}_{P_j}^0\left[e^{ux_i(0^-)}\right] \\ &= \beta_j t\,\mathbb{E}\left[e^{ux_i}\right] \\ &= \beta_j t L_i(u),\end{aligned} \tag{6}$$

where the second equality is because P_j is an independent process. For the last term, we use Eq. (4) and Eq. (5) successively and get

$$\begin{aligned}\mathbb{E}\left[\int_0^t \left(1-e^{ux_i(s)}\right) N_i(\mathrm{d}s)\right] &= \beta_i t\,\mathbb{E}_{N_i}^0\left[1-e^{ux_i(0^-)}\right] \\ &= \beta_i t - \beta_i t\,\mathbb{E}_{N_i}^0\left[e^{ux_i(0^-)}\right] \\ &= \beta_i t - t\,\mathbb{E}\left[h_i e^{a_i x_i} e^{ux_i}\right] \\ &= \beta_i t - t h_i L_i(u+a).\end{aligned} \tag{7}$$

Now we can substitute Eq. (3), Eq. (6) and Eq. (7) into Eq. (2) and the desired DDE will be recovered once canceling the common factor t.

B Simulation method

In the main text, we compare the theoretical and simulated results of the LER model throughout. The simulated results are obtained from an event driven method for simulating spiking neuron network [2,3]. Here, we outline the procedure of this method.

For the exponential firing neuron with internal variable x, the time till next spike is distributed according to the following complementary cumulative distribution function (survival function):

$$\mathbb{P}(T>t) = \exp\left\{-\int_0^t h e^{axe^{-s/\tau}}\,\mathrm{d}s\right\}. \tag{8}$$

When $x=0$, the distribution Neuron(τ,h,a,x) reduces to Exp(h). When $x\neq 0$, we can integrate Eq. (8) and obtain explicitly

$$\mathbb{P}(T>t) = \exp\left\{h\tau\left[\mathrm{Ei}(axe^{-t/\tau}) - \mathrm{Ei}(ax)\right]\right\}, \tag{9}$$

where $\mathrm{Ei}(x) = -\int_{-x}^{\infty} e^{-t}/t\,\mathrm{d}t$ is the exponential integral function. Then we apply inverse transform sampling technique by sampling $u\sim$ Uniform$(0,1)$ and solving $\mathbb{P}(T>t)=u$ for t. Using the fact that $\mathrm{Ei}(x)=\mathrm{li}(e^x)$, where $\mathrm{li}(\cdot)$ is the logarithmic integral function, we have

$$t = -\tau\ln\left\{\frac{1}{ax}\ln\left[\mathrm{li}^{-1}\left(\mathrm{Ei}(ax)+\frac{1}{h\tau}\ln u\right)\right]\right\}. \tag{10}$$

Note that the branch of $\mathrm{li}(\cdot)$ function should be determined by the sign of x: when $x<0$, we choose the left branch and when $x>0$, we choose the right branch.

At each iteration step, we sample a random number t representing waiting time till the next event for each neuron. Among all sampled waiting time: $t_{\text{neuron},1}, t_{\text{neuron},2}, \cdots$, we choose the smallest one as the time of occurrence of the next event. Suppose this event has waiting time t_{next}. For all the neurons, we multiply an exponential relaxation factor $e^{-t_{\text{next}}/\tau}$ to the internal variables. Then, for all the neuron that are connected to the source neuron of this event, we add the corresponding connection strength to the internal variables. Namely,

$$x_i \leftarrow x_i e^{-t_{\text{next}}/\tau} + \mu_{\text{source}\to i}. \tag{11}$$

We collect all necessary information at each iterative step and repeat this process until certain criteria are satisfied (event counts, simulated time, etc.).

C Calculation for higher moments

In the main text, we compute the second moments of the intensity λ and internal variable x of our model. The preservation of higher moments is one of the benefits of our model. In fact, we can compute the moments of arbitrary orders, which we formulate here.

Recall that the moments of λ and x are related in LER models:

$$M_n(\lambda) = h^n \sum_{k=0}^{\infty} \frac{(an)^k}{k!} M_k(x)\,. \tag{12}$$

We formulate the equations for the higher moments of x and those of λ can be computed by using truncated Eq. (12). From the MGF $L(u) = \mathbb{E}\left[e^{ux}\right]$, the moments of x can be written as $M_k(x) = L^{(k)}(0)$. Since $H(u) = (L(u+a) - L(a))/u$ and H has the following series expansion

$$H(u) = \frac{\beta}{h} \sum_{m=0}^{\infty} (-h\tau)^m Q_m(u)\,, \tag{13}$$

we arrive at

$$M_k(x) = \left[uH(u)\right]^{(k)}\Big|_{u=-a} = \frac{\beta}{h} \sum_{m=0}^{\infty} (-h\tau)^m \left[uQ_m(u)\right]^{(k)}\Big|_{u=-a}\,. \tag{14}$$

Eq. (14) is valid for $k \geq 1$ and can be computed using Padé approximants summation in a similar fashion.

D Different approximation methods

In the main text, we compare our theoretical calculation with the no-reset approximation and TMF approximations. Here, we summarize these different approximation methods.

D.1 No post-spiking reset (low rate approximation)

When the input rate is low, the neuron integrates the received inputs slowly and also spikes sparsely. The neuron relaxes on a faster time scale and prevents the internal variable from going to infinity and the reset mechanism does not play significant roles. In this case, the DDE reduces to the ODE $uL'(u) = \tau V(u)L(u)$. With the normalization condition $L(0) = 1$ we can solve $L(u) = q(u)/q(0)$, where $q(u) = \exp\left(\tau \int_a^u V(v)/v \,\mathrm{d}v\right)$.

The n-th moment of the intensity λ is given by

$$\mathbb{E}\left[\lambda^n\right] = h^n L(ka) = h^n \frac{q(na)}{q(0)}. \tag{15}$$

Specifically, the mean and variance of λ are

$$\mathbb{E}\left[\lambda\right] = \frac{h}{q(0)},\ \mathbb{V}\left[\lambda\right] = \frac{h^2}{q^2(0)}\left[q(2a)q(0) - 1\right]. \tag{16}$$

The k-th cumulant ($k \geq 1$) of x can be calculated:

$$\kappa_k = \left[\ln(L(0))\right]^{(k)} = \frac{1}{k}V^{(k)}(0) = \frac{\tau}{k}\sum_{\text{input } j}\beta_j\mu_j^k. \tag{17}$$

Specifically, the mean and variance of x are

$$\mathbb{E}\left[x\right] = \tau\sum_{\text{input } j}\beta_j\mu_j,\ \mathbb{V}\left[x\right] = \frac{\tau}{2}\sum_{\text{input } j}\beta_j\mu_j^2. \tag{18}$$

D.2 TMF limits without relaxation

In the TMF limits, the neuron receives infinitesimal signal deliveries from infinitely many inputs. Thus, the accumulation of the neuronal internal variable can be viewed as an integration. When the intensity λ integrates to one, the neuron fires deterministically:

$$\int_0^{t_{\text{fire}}}\lambda(t)\,\mathrm{d}t = \int_0^{t_{\text{fire}}} he^{ax(t)}\,\mathrm{d}t = 1. \tag{19}$$

Further, we assume that when the spiking is very frequent, the relaxation does not play significant roles. Then we have $x(t) = \boldsymbol{\beta}\cdot\boldsymbol{\mu}\,t$, where $\boldsymbol{\beta}\cdot\boldsymbol{\mu} = \sum_{\text{input } j}\beta_j\mu_j$. Together with Eq. (19), we can solve t_{fire}:

$$t_{\text{fire}} = \frac{1}{a\boldsymbol{\beta}\cdot\boldsymbol{\mu}}\ln\left(1 + \frac{a\boldsymbol{\beta}\cdot\boldsymbol{\mu}}{h}\right). \tag{20}$$

The n-th moment of the intensity λ is given by

$$\mathbb{E}\left[\lambda^n\right] = \frac{1}{t_{\text{fire}}}\int_0^{t_{\text{fire}}} h^n e^{nax(t)}\,\mathrm{d}t = \frac{h^n(e^{na\boldsymbol{\beta}\cdot\boldsymbol{\mu}t_{\text{fire}}} - 1)}{na\boldsymbol{\beta}\cdot\boldsymbol{\mu}\,t_{\text{fire}}}. \tag{21}$$

Specifically, the mean and variance of λ are

$$\mathbb{E}\left[\lambda\right] = \frac{1}{t_{\text{fire}}},\ \mathbb{V}\left[\lambda\right] = \frac{h^2(e^{2a\boldsymbol{\beta}\cdot\boldsymbol{\mu}t_{\text{fire}}} - 1)}{2a\boldsymbol{\beta}\cdot\boldsymbol{\mu}\,t_{\text{fire}}} - \frac{1}{t_{\text{fire}}^2}. \tag{22}$$

The k-th moment of x can be calculated:

$$\mathbb{E}\left[x^k\right] = \frac{1}{t_{\text{fire}}}\int_0^{t_{\text{fire}}}(\boldsymbol{\beta}\cdot\boldsymbol{\mu})^k\,t^k\,\mathrm{d}t = \frac{(\boldsymbol{\beta}\cdot\boldsymbol{\mu})^k}{k+1}t_{\text{fire}}^k. \tag{23}$$

Specifically, the mean and variance of x are

$$\mathbb{E}\left[x\right] = \frac{\boldsymbol{\beta}\cdot\boldsymbol{\mu}}{2}t_{\text{fire}},\ \mathbb{V}\left[x\right] = \frac{(\boldsymbol{\beta}\cdot\boldsymbol{\mu})^2}{12}t_{\text{fire}}^2. \tag{24}$$

D.3 TMF limits with relaxation

Similarly to the previous case, but when the spiking is not frequent and relaxation cannot be neglected, we have $x(t) = \boldsymbol{\beta} \cdot \boldsymbol{\mu} \tau (1 - e^{-t/\tau})$. Integrate Eq. (19) and we can solve t_{fire} from the following equation

$$h\tau e^{a\boldsymbol{\beta}\cdot\boldsymbol{\mu}\tau} \left[\text{Ei}(-a\boldsymbol{\beta}\cdot\boldsymbol{\mu}\tau) - \text{Ei}\left(-a\boldsymbol{\beta}\cdot\boldsymbol{\mu}\tau e^{-t_{\text{fire}}/\tau}\right) \right] = 1. \tag{25}$$

This can be done similarly as solving Eq. (9) in Appendix B.

The n-th moment of intensity λ is given by

$$\begin{aligned} &\mathbb{E}\left[\lambda^n\right] \\ =&\frac{h^n \tau e^{na\boldsymbol{\beta}\cdot\boldsymbol{\mu}\tau}}{t_{\text{fire}}} \left[\text{Ei}(-na\boldsymbol{\beta}\cdot\boldsymbol{\mu}\tau) - \text{Ei}\left(-na\boldsymbol{\beta}\cdot\boldsymbol{\mu}\tau e^{-\frac{t_{\text{fire}}}{\tau}}\right) \right]. \end{aligned} \tag{26}$$

Specifically, the mean and variance of λ are

$$\mathbb{E}\left[\lambda\right] = \frac{1}{t_{\text{fire}}},\ \mathbb{V}\left[\lambda\right] = \mathbb{E}\left[\lambda^2\right] - \frac{1}{t_{\text{fire}}^2}. \tag{27}$$

The k-th moment ($k \geq 1$) of x can be calculated:

$$\mathbb{E}\left[x^k\right] = \frac{(\boldsymbol{\beta}\cdot\boldsymbol{\mu}\tau)^k}{t_{\text{fire}}} \int_0^{t_{\text{fire}}} (1 - e^{-t/\tau})^k \, \mathrm{d}t. \tag{28}$$

Specifically, the mean of x is

$$\mathbb{E}\left[x\right] = \frac{\boldsymbol{\beta}\cdot\boldsymbol{\mu}\tau}{t_{\text{fire}}} \left[t_{\text{fire}} + \tau(e^{-t_{\text{fire}}/\tau} - 1) \right] \tag{29}$$

and the variance of x is

$$\begin{aligned} &\mathbb{V}\left[x\right] \\ =&\frac{(\boldsymbol{\beta}\cdot\boldsymbol{\mu})^2\tau^3 \left(1 - e^{-\frac{t_{\text{fire}}}{\tau}}\right)\left(e^{-\frac{t_{\text{fire}}}{\tau}}(t_{\text{fire}} + 2\tau) + t_{\text{fire}} - 2\tau\right)}{2t_{\text{fire}}^2}. \end{aligned} \tag{30}$$

E Prediction of dynamical transition rates

In the main text, we present some results about the prediction of transition rates in the bistable networks. We supplement more technical details in this appendix.

The Eyring-Kramer's law [4, 5] describes the transition time of a Brownian particle in a double-well potential $V(x)$:

$$\mathbb{E}\left[T_{\text{transition}}\right] \sim \frac{2\pi}{\sqrt{V''(x^*)|V''(z^*)|}} e^{[V(z^*) - V(x^*)]/\epsilon}, \tag{31}$$

where x^* is the starting local minimum of $V(x)$, z^* is the unstable local maximum of $V(x)$ and ϵ is the noise parameter of the Brownian system. To apply this formula in the case of our bistable networks, we need some notion of potential energy. To this end, we note that such Brownian system admits an invariant probability distribution satisfying

$$p(x) = \frac{1}{Z} e^{-V(x)/\epsilon}, \tag{32}$$

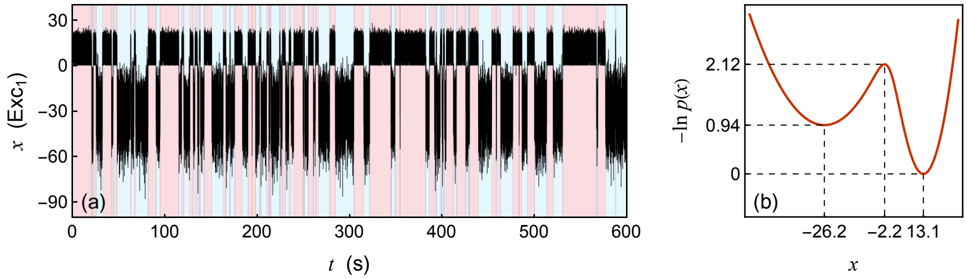

Fig A. Bistable networks. Panel (a): The simulated time series of the internal variable x of an representative excitatory neuron in the Group 1. Panel (b): The function $-\ln p(x)$ using quantities from RMF calculation. This represents the potential energy landscape of the system. Parameters: $K_{\text{total}} = 40$, $\mu_e = 1.5$, $\mu_i = 10$, $h = 1\,\text{Hz}$, $a = \ln(100)/20 \approx 0.23$, $\tau = 10\,\text{ms}$.

where Z is a normalization constant. By taking the logarithm on both sides of Eq. (32), we can relate the potential energy with the probability distribution up to an arbitrary constant $-\ln Z$ and a scaling parameter ϵ:

$$\frac{V(x)}{\epsilon} = -\ln p(x) - \ln Z. \tag{33}$$

Our goal is to estimate the probability distribution $p(x)$. In Fig. A(a), we show the time series of the internal variable x of a representative excitatory neuron in Group 1. We observe that the internal variable x in the bistable state naturally admits a bimodal distribution. This suggests approximating the distribution $p(x)$ with the following mixture of two normal distributions:

$$p(x) = \frac{1}{2}\frac{1}{\sqrt{2\pi}\sigma_{\text{u}}} e^{-(x-x_{\text{u}})^2/\sigma_{\text{u}}^2} + \frac{1}{2}\frac{1}{\sqrt{2\pi}\sigma_{\text{d}}} e^{-(x-x_{\text{d}})^2/\sigma_{\text{d}}^2}. \tag{34}$$

In Eq. (34), $x_{\text{u(d)}}$, $\sigma_{\text{u(d)}}$ are the mean and standard deviation of x in the up(down) state computed by our RMF calculation. We expect the mixture weights to be both 1/2 because of the complete symmetry of the network. It is worth noting that this distribution is determined by the theoretical RMF calculation without using any information from simulation. We plot the function $-\ln p(x)$ (offsetting the lowest valley at zero) in Fig. A(b). We observe that, indeed, such estimation yields a system with a double-well potential and is valid for the application of Eyring-Kramer's law.

We can rewrite the transition time Eq. (31) with $p(x)$ using Eq. (33). Namely, we have

$$\mathbb{E}\left[T_{\text{transition}}\right] \sim \frac{1}{\epsilon} \frac{2\pi}{\sqrt{-[\ln p(x^*)]''|[-\ln p(z^*)]''|}} \frac{p(x^*)}{p(z^*)}. \tag{35}$$

Note that the constant $-\ln Z$ cancels out and the noise parameter ϵ serves as a scaling coefficient. Before proceeding, we should address two questions:

First, we need to determine the starting state x^*. We claim that, in the bistable network under consideration, the transition is driven by the down-to-up switch. This is supported by the simulation. Fig. B(a) shows the excitatory-cluster-averaged evolution paths $(\overline{x_{\text{Exc}_1}}(t), \overline{x_{\text{Exc}_2}}(t))$ over the entire simulation period, containing 500000 spiking events and 100 transition events. From these trajectories, we observe that the system almost always goes through the top-right corner of the diagram when switching state. This strongly suggests that the system enters a transient up-up coexisting state during switching. Fig. B(b) and (c) show two examples of such switching by plotting the evolution paths $(x_{\text{Exc}_1}(t), x_{\text{Exc}_2}(t))$ of a pair of representative neurons in two excitatory

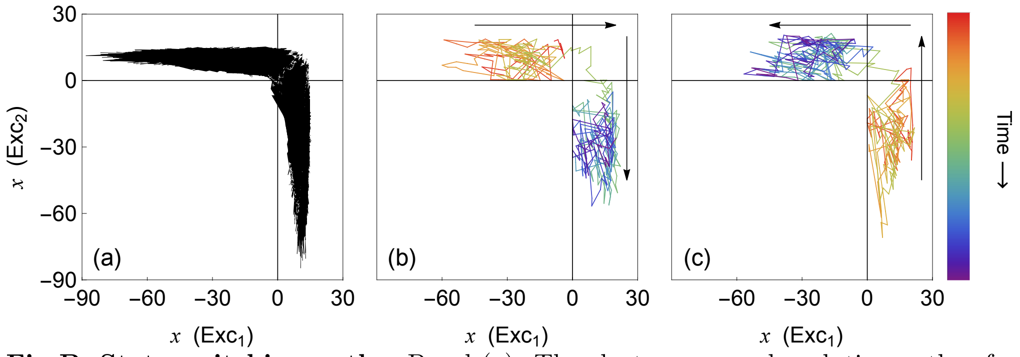

Fig B. State switching paths. Panel (a): The cluster-averaged evolution paths of $(\overline{x_{\text{Exc}_1}}(t), \overline{x_{\text{Exc}_2}}(t))$ over the entire simulation period of 500000 spiking events in total. During this simulation period, 100 transitions are recorded. Panel (b) and (c): Two example paths of representative neuron pairs $(x_{\text{Exc}_1}(t), x_{\text{Exc}_2}(t))$ near transitions in two opposite switching directions. The color indicates the direction of time. Parameters: $K_{\text{total}} = 40$, $\mu_e = 1.5$, $\mu_i = 10$, $h = 1\,\text{Hz}$, $a = \ln(100)/20 \approx 0.23$, $\tau = 10\,\text{ms}$.

clusters. From Fig. B(b), we see that $(x_{\text{Exc}_1}(t), x_{\text{Exc}_2}(t))$ goes through (down, up)→(up, up)→(up, down); Fig. B(c) shows an case of opposite switching direction, where $(x_{\text{Exc}_1}(t), x_{\text{Exc}_2}(t))$ goes through (up, down)→(up, up)→(down, up). Thus, the onset of a transition is driven by a down-to-up switch in both cases and x^* should be the valley corresponding to the down state.

The second question is to determine the noise parameter ϵ of the system. As a preliminary study, we numerically find the following formula describes the dependence of ϵ on μ_e and μ_i:

$$\epsilon = \epsilon_0 \left(\mu_e \mu_i\right)^{-3/2}, \tag{36}$$

where ϵ_0 is a constant depending on all other network parameters, i.e., network size K, neural spontaneous spiking rate h, exponential firing exponent a. Further investigation is needed to both justify Eq. (36) and the form of ϵ_0. In this work, we determine ϵ_0 by fitting the simulated data.

References

1. Baccelli F, Brémaud P. The Palm Calculus of Point Processes. In: Elements of Queueing Theory. Springer; 2003. p. 1–74.

2. Matthes K. Zur Theorie der Bedienungsprozesse. In: Trans. Third Prague Conf. Information Theory, Statist. Decision Functions, Random Processes (Liblice, 1962). Publ. House Czech. Acad. Sci., Prague; 1964. p. 513–528.

3. Taillefumier T, Touboul J, Magnasco M. Exact Event-Driven Implementation for Recurrent Networks of Stochastic Perfect Integrate-and-Fire Neurons. Neural Computation. 2012;24(12):3145–3180.

4. Kramers HA. Brownian motion in a field of force and the diffusion model of chemical reactions. Physica. 1940;7(4):284–304.

5. Eyring H. The activated complex in chemical reactions. The Journal of Chemical Physics. 1935;3(2):107–115.
