# Supplementary material for: Metastable spiking networks in the replica-mean-field limit
Source: PLoS Comput Biol. 2022 Jun 17;18(6):e1010215. doi: 10.1371/journal.pcbi.1010215 (PMC9246178; doi:10.1371/journal.pcbi.1010215)
Supplement: S1 Fig — The distribution of the internal variable x of a single neuron subjected to various different types of inputs. The detailed input parameters are listed above. Parameters: h = 1 Hz, a = 0.1, τ = 10 ms. (PDF) [file pcbi.1010215.s002.pdf]

S2 Figure: Distribution of the internal variable x.

Luyan Yu[1], Thibaud Taillefumier[2, 3*]

1 Department of Physics, University of Texas at Austin, Austin, Texas, USA
2 Department of Mathematics, University of Texas at Austin, Austin, Texas, USA
3 Department of Neuroscience, University of Texas at Austin, Austin, Texas, USA

* ttaillef@austin.utexas.edu

Fig A is the simulated density function $p(x)$ of the internal variable x of a single neuron subjected to different inputs. The simulations are performed until 500000 spiking events are accumulated. The detailed input parameters are listed below:

- In panel (a), $\beta_e = 1\,\mathrm{kHz}$, $\mu_e = 1$; in panel (b), $\beta_e = 1\,\mathrm{kHz}$, $\mu_e = -1$.
- In panel (c), $\beta_e = 1\,\mathrm{kHz}$, $\mu_e = 5$; in panel (d), $\beta_e = 1\,\mathrm{kHz}$, $\mu_e = -5$.
- In panel (e), $\beta_e = 10\,\mathrm{Hz}$, $\mu_e = 1$; in panel (f), $\beta_e = 10\,\mathrm{Hz}$, $\mu_e = -1$.
- In panel (g), $\beta_e = 0.5\,\mathrm{kHz}, \beta_i = 0.1\,\mathrm{kHz}$, $\mu_e = 1, \mu_i = -1$;
  in panel (h), $\beta_e = 0.1\,\mathrm{kHz}, \beta_i = 0.5\,\mathrm{kHz}$, $\mu_e = 1, \mu_i = -1$.
- In panel (i), $\beta_e = 1\,\mathrm{kHz}, \beta_i = 1\,\mathrm{kHz}$, $\mu_e = 1, \mu_i = -1$;
  in panel (j), $\beta_e = 10\,\mathrm{Hz}, \beta_i = 10\,\mathrm{Hz}$, $\mu_e = 1, \mu_i = -1$.
- In panel (k), $\{\beta_j\}_{j=1}^{100}$ uniformly sampled from range $[10\,\mathrm{Hz}, 100\,\mathrm{Hz}]$, $\{\mu_j\}_{j=1}^{100}$ uniformly sampled from range $[-1, 1]$;
  in panel (l), $\{\beta_j\}_{j=1}^{100}$ uniformly sampled from range $[100\,\mathrm{Hz}, 1000\,\mathrm{Hz}]$, $\{\mu_j\}_{j=1}^{100}$ uniformly sampled from range $[-1, 1]$.

Note that the vertical axes of the figures are in log scale. These histograms show that the tails of the density function $p(x)$ are decaying in a exponential manner in most cases. (In the cases of pure inhibition, it is at least as fast as exponential decay.)

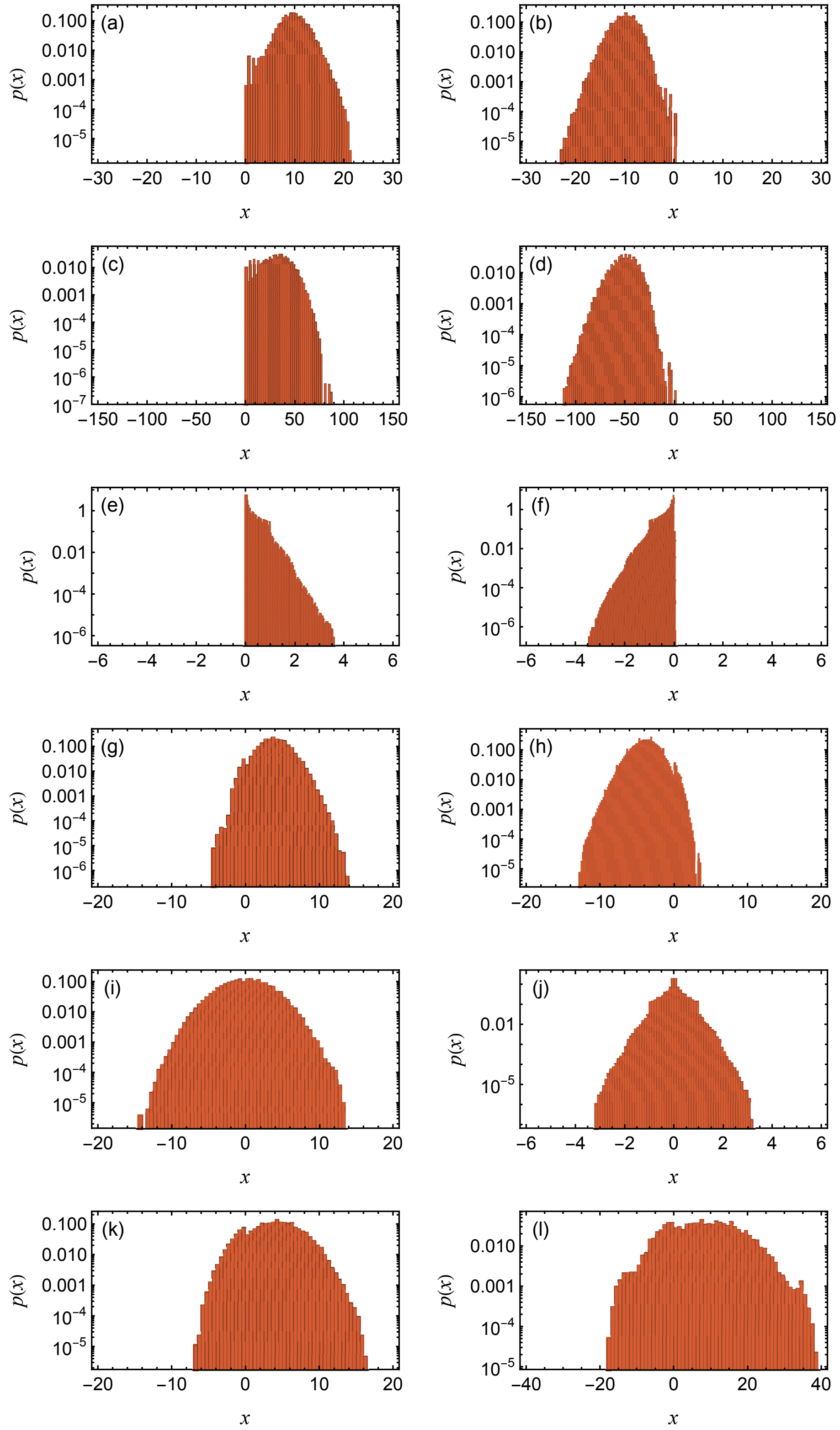

Fig A. Distribution of the internal variable x. The distribution of the internal variable x of a single neuron subjected to various different types of inputs. The detailed input parameters are listed above. Parameters: $h = 1\,\mathrm{Hz}$, $a = 0.1$, $\tau = 10\,\mathrm{ms}$.
