# Supplementary material for: Metastable spiking networks in the replica-mean-field limit
Source: PLoS Comput Biol. 2022 Jun 17;18(6):e1010215. doi: 10.1371/journal.pcbi.1010215 (PMC9246178; doi:10.1371/journal.pcbi.1010215)
Supplement: Attachment — Submitted filename: Response Letter and Description of Changes.pdf [file pcbi.1010215.s003.pdf]

Revision of Manuscript PCOMPBIOL-D-21-01383

Luyan Yu and Thibaud Taillefumier

Dear Editors,

Thank you for taking the time to review our manuscript titled "Metastable networks in the replica-mean-field limit". This letter is a response to the reviewers' comments and a description of the changes to the manuscript. We appreciate your support.

Sincerely
Luyan Yu
Thibaud Taillefumier

Response to Reviewer 1

Major comments:

1. The lack of novel biologically-relevant insights in the present manuscript renders it somewhat unsatisfying. While the technical component of this work presents a well-defined incremental advance relative to prior work by the second author (notably Refs. [25] and [46]), the main result is an approximate numerical framework which does not in of itself yield substantial insight.

We appreciate the reviewer's comments and we agree that our biological motivation was not stated clearly enough. To address the reviewer's point, we rewrote the introduction of the manuscript to showcase the key motivation for our approach. This motivation is to account for the role that individual inputs can play in shaping the spiking output of a neuron. This is by contrast with most commonly used computational frameworks, which either ignore such individual impacts (e.g., classical mean-field models) or resist any analytical treatments. For these models, it is unclear, or at the very least very computationally expensive, to estimate the parametric dependences of the neural activity on the key parameters specifying neural circuits. Such parameters include the typical finite number of neuronal inputs, as well as the sizes of these inputs.

Here, we would like to complement the points made in the introduction by arguing that being able to relate neural variability and network parameters quantitatively is of great biological significance. We hope to achieve this by discussing experimental results from the Priebe and Seidemann labs at UT Austin, which we also mention in response to Reviewer 2. We do not include these results in the main manuscript as they will form the basis of a forthcoming paper by the Priebe and Seidemann labs. These results are presented in Fig. R1. A, where one can see that the mean and the variance of the membrane voltage of V1 neurons increase with stimulus onset in awake behaving monkeys. More

generally, measurement in the Priebe and Seidemann labs shows that the mean voltage and the voltage variability jointly increase with the stimulus drive. This is contrary to similar measurements in other mammals, where variability quenching has been reported in response to stimulus presentation.

We claim that a joint increase of the mean voltage and of the voltage variability cannot be explained within the confines of classical mean-field approaches. Indeed, classical thermodynamic limits ($1/N$ scaling) can only capture the proper mean response at the cost of a vanishing variability due to usual population averaging. Balanced limits ($1/\sqrt{N}$ scaling) can reproduce the proper variability dependence on the stimulus contrast but only at the cost of producing a nearly constant mean response. We believe both these mean-field approaches fails to appropriately capture the neural response because they neglect finite-size effects. To remedy this point, one can apply the RMF framework to a realistic conductance-based neural network. Such treatment shows that all aspects of the measured neuronal response can be recovered if the drive is primarily mediated by a few large synapses (< 50) with effective weights falling within a biologically plausible range ($w < 0.1$ms) (see Fig. R1. B). By biologically feasible, we mean that a single spiking event does not lead to

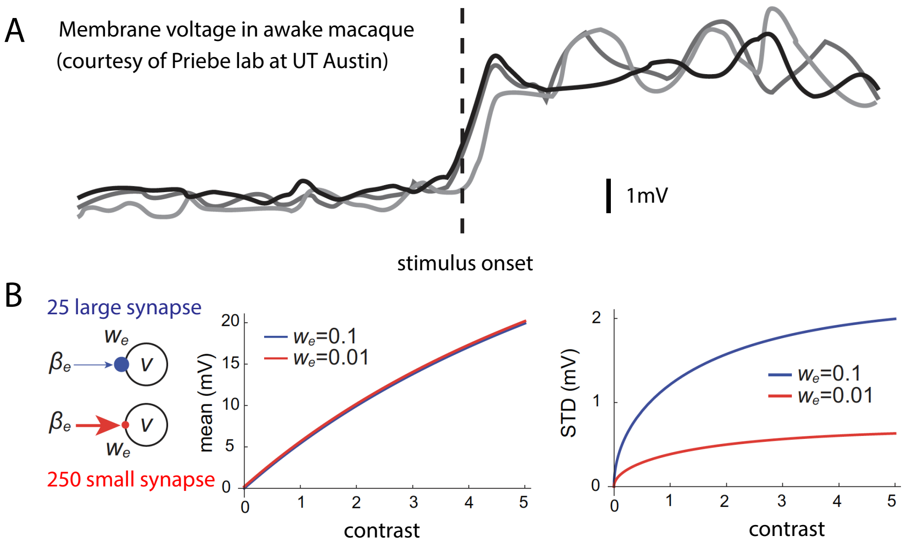

Fig. R1. **Measurement of membrane voltage in awake macaque.** Panel A: Sample measurements of the voltage time series around the stimulus onset. (Data from private communication with Priebe lab at UT Austin.) Panel B: The RMF method can capture the correct behavior of increasing variability.

larger than 0.5 mV fluctuations in the membrane voltages, as verified experimentally. This simple analysis argues that finite-size effects alone can account for the joint variation of the voltage mean and of the voltage variability.

The question addressed above is simple enough to be addressed in the RMF limit of a biophysically detailed model, such as a conductance-based one. When the questions at stake bare on collective aspects of the neural dynamics, such as in metastable dynamics, we expect the detailed structure of the network to play the central role. In the context of metastable dynamics, we believe that our proposed EGL models exhibit the satisfactory level of biological relevance. In this regard, note that

EGL neurons can produce the appropriate scaling of mean and variability, as opposed to other mean-field approaches. More generally, we would also like to point out that our analysis can be carried out for arbitrary topology and does not rely on any form of quenched-disorder hypothesis. We believe this to be quite a unique feature of our approach.

I am not convinced that the solution method---which relies upon a combination of relatively standard perturbative methods---is sufficiently innovative to justify publication without further evidence of its broad utility. The scientific contribution of the present work would be much clearer if the authors could show more clearly that their methods yield novel biologically-relevant insights beyond the possibility of detecting the transition to bistability.

We acknowledge that the basic ideas of the RMF framework were presented in two prior publications. However, we would like to kindly point out that these ideas were communicated in journals that target a mathematical audience and in a language that, we fear, is not accessible to a nontechnical audience. The submitted work represents our first attempt to communicate the ideas of the RMF approach beyond the mathematical community. Moreover, in this work, our goal is also to expand the RMF approach in a way that is both accessible and relevant to a broader audience of neuroscientists, with a focus on biophysically relevant phenomena of practical interest, such as metastable neural dynamics.

We also agree that some aspects of our approach can be thought as standard. For instance, the use of Padé approximants to extend the domain of convergence of a series is well documented in the physics literature. However, we are not aware of any analytical method that reduces the problem of characterizing the typical state of a spiking network to a nonlocal (delay) differential problem baring on a moment-generating function. This nonlocal form stems from taking into account the feedback that a neuron's own spiking exerts on its dynamics via reset. We are not aware of other studies that formally identified the inclusion of a reset mechanism as a singular perturbation problem. Such an identification allowed us to rigorously discuss the various spiking regimes of an EGL neuron in relation to the three timescales associated with relaxation, spontaneous firing, and output firing (feedback). We believe this discussion to be independent of modeling details as long as a reset mechanism is included.

One clear way for the authors to enhance the impact of this paper would be to at least partially realize their ``hope [of leveraging the] RMF framework to estimate key dynamical properties of metastable dynamics'' (lines 858-859). I found it somewhat frustrating that the authors reported a preliminary result that would clearly support the idea that their framework can provide useful insights towards the stated goal of understanding metastability (namely, that ``the static approach of the RMF framework is predictive of the transition rates,'' lines 866-867) in the Discussion. This leaves the reader with the impression that the present work is somewhat incomplete. I would therefore suggest that the authors take the time to treat this point carefully, and then submit a manuscript containing a more scientifically complete story. Alternatively, they might consider submitting the present manuscript to a more specialized journal.

We agree with the reviewer that a clear demonstration of the merits of the RMF approach in the context of metastability would improve our manuscript. We address this point in this revised manuscript by adding a figure (Fig. 14) demonstrating that transition rates can be predicated on the basis of our RMF approach. Specifically, we leverage the knowledge of the mean and standard deviation in the RMF limit to infer an effective energy landscape using a mixture of normal distributions. We then utilized this energy landscape to exhibit a modified Eyring-Kramer's law, which predicts the expected dynamical transition time of the bistable systems. These predictions are computed solely based on RMF calculations up to a scaling parameter, whose functional form we hope to elucidate in future studies. We provide the details of our derivation of the proposed Eyring-Kramer's law in Appendix S1. We also justify the validity of our one-dimensional effective energy landscape by discussing the correlation structure of the bistable dynamics (see main text and a new figure (Fig. 12)) and by identifying the escape process from down state as the main determinant of the switching time scale (see Appendix S1). As mentioned at the end of the main text, we hope to be able to systematically derive such Eyring-Kramer's laws via large-deviation principles in the future.

2. Some revision to the prose would be required in order to make the paper accessible to PLoS Comput. Biol.'s broad audience. While this referee certainly appreciates the difficulties inherent in crafting a manuscript that is both technically rigorous and broadly accessible, the balance struck by the current version could be improved. Given the fact that the second author's prior publications on the RMF method provide the requisite technical details rigorously, one option would be for the authors to take this opportunity to give a more pedagogical introduction to these ideas before detailing the current technical advance. The authors might alternatively consider re-organizing the manuscript to emphasize biologically-relevant results before describing some of the methodological details in full.

Even for a reader relatively well-versed in the required mathematics and neuroscience, the readability of the manuscript is compromised by the distracting frequency of typos and grammatical errors.

We are thankful for the reviewer's help in improving the manuscript. We followed the reviewer's first suggestion. Specifically:

1 – We extensively reworked the introduction to clarify the main biological motivation, i.e., quantifying the role of finite-size effects, with a special focus on metastable dynamics. We also clarified the limitations of existing classical mean-field approach and presented a nontechnical, high-level description of the RMF approach. Finally, we added a paragraph discussing the merits of our methodology as well as its originality.

2 – We gave a more pedagogical introduction to the RMF method applied to stochastic-intensity based models in a subsection of "*Modeling and Theory*" called: "*General approach and dynamics of interest*". This subsection contains three paragraphs:

A – In the first paragraph, we discuss the essential tenets of the RMF method for generic spiking networks, avoiding technical modeling assumptions. We also intuitively justify how the key idea of randomizing interactions across replicas naturally leads to the simplifying Poisson hypothesis.

B – In the second paragraph, we apply the RMF method on the simplest example of intensity-based models, i.e., the linear Hawkes processes. In doing so, we especially emphasize the distinction between RMF and classical mean-field approaches.

C – In the third paragraph, we introduce the EGL dynamics of interest as generalized Hawkes dynamics and discuss the two involved modeling complications: the nonlinearity of the rate function and the finite memory of neuronal processing due to reset. We also stress the feedback role of including a post-spiking reset.

3 – We apologize for the typos that distracted the reading. We corrected the typos and grammatical errors in the revised manuscript. We thank the reviewer for carefully reading our manuscript.

3. As the RMF method results in self-consistent equations that must ultimately be approximated and solved numerically through a perturbative scheme, it is important that the authors provide sufficient justification for why this method is advantageous relative to direct Monte Carlo simulation. Some examples of where this point should be reified are as follows:

We agree with the reviewer that this is an important aspect of our approach and that some details were lacking in the original manuscript. We have addressed these points in the main manuscript (see our response to "a." and "b." below for a summary of the changes).

a. Lines 433-434: ``Bear in mind that in all cases, our computational results are obtained incomparably faster than those estimated via Monte-Carlo simulations." This claim---particularly given the hyperbolic descriptor ``incomparably"---should be supported with explicit runtime statistics, as well as a detailed description of the computational resources used to generate the presented results.

In the revised version, we added a section called "*Computational efficiency*" to demonstrate the numerical efficiency of our method. The results are presented in Fig. 4 under that section. Here, for the sake of conciseness, we summarize our main numerical observations. Generating accurate estimates for spiking rate via Monte-Carlo method generally requires a large number of spiking events. We numerically find that achieving a reasonable criterion for accuracy (5% relative error) requires several hundreds of thousands spiking events. This represents a computational cost at least two orders of magnitudes higher than that of the RMF method. Importantly, note that the comparatively high computational Monte-Carlo cost is obtained for efficient event-driven simulation scheme (Gillespie methods), as opposed to naïve schemes relying on discrete mesh-grid time.

b. Lines 678-679: ``We conclude by utilizing our RMF computational approach to exhibit behaviors that are otherwise challenging to obtained via simulations." Please provide a more detailed argument to justify this claim.

As mentioned above, it is also important---particularly given the readership of PLoS Comput. Biol.---to argue that this method has strong potential to generate novel biological insights that could not easily be gleaned from Monte Carlo simulations.

We believe that an example of challenging numerical tasks for Monte-Carlo methods is determining the critical line marking the start of the metastable region in Fig 13(b). We find that determining such

a critical line via Monte-Carlo methods takes more than 3 weeks using a dichotomic search procedure, while enforcing the same 5%-relative-error constraint as mentioned in response to point "a.". By contrast, our RMF method only takes 7 minutes. To further support our claim of efficiency, we plot in Fig. R2 the computational times for both the RMF method and the numerical simulation as a function of the cluster sizes of the bistable network. Fig. R2 shows that the computational time of the simulation increases with the cluster size as a power law with exponent about 2.5, whereas our RMF method is strongly sublinear with respect to cluster size. This shows concretely that our RMF method offer a substantial computational benefit. We hope that the above response to reviewer's point "b" will be enough. However, should the reviewer ask for it, we are also willing to add the additional comments to the main manuscript.

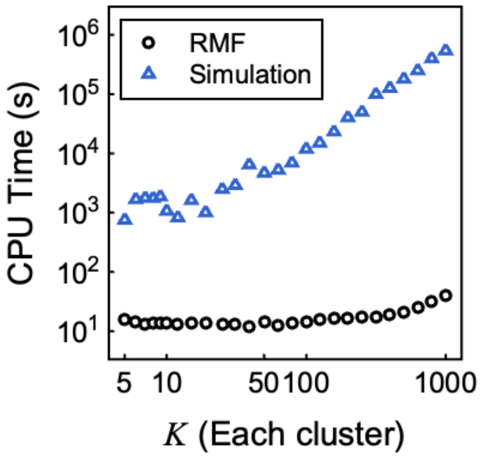

Fig. R2. **Computational time for generating Fig 13(b) in the manuscript.** In this figure, we show the comparison of the computational time used by simulation and RMF calculation for different cluster sizes. For simulation, we run the simulation until an average of 400 spiking events for each neuron are accumulated. This process is repeated for 32 times.

Minor Comments:

1. Figure 1 appears to be identical to Figure 4 of Ref. [46] (Baccelli and Taillefumier, SIAM J. Applied Dynamical Systems 2021); reproduction should be noted.

Thank you for the suggestion. The reproduction is now acknowledged.

2. Lines 277-278: Can you provide evidence for this conjecture?

We can indeed provide numerical evidence supporting this claim. Specifically, in the newly added supplementary material S2, we estimated the density functions of the internal variable x from simulation data. When displayed in log-log plots, we found that the tail behavior of the PDFs exhibits at least exponential decay for all choices of parameters, including for the cases of strong excitatory drive, strong inhibitory drive and balanced drive.

3. Figures 4-10: Please specify sample sizes in each figure caption and indicate precisely how error bars are computed. Some information is given in the caption for Figure 4, but it is not sufficient.

We added the requested information about computations in each figure.

4. Lines 639-642: Please elaborate on this point.

After further inquiry, we are able to provide the reviewer with a more detailed explanation for why our RMF approach is able to capture bistable dynamics while neglecting correlations. This explanation follows from Fig. 12, which elucidates the correlation structure of the bistable dynamics in the original system. It turns out that during the full dynamics, correlations primarily emerge due to stochastic switches. Accordingly, conditioning on a group of neurons to be up or down leads to a drastic simplification of the correlation structure. This simplified correlation structure happens to be dominated by the feedforward drives of up neurons. Regimes of activity that are dominated by feedforward drive are generally well approximated by the RMF setting. This explains why the multistable RMF limit is able to approximate each conditional regime satisfactorily. These observations are included in a new paragraph of the main text (page 28).

5. Line 750: I cannot follow the usage of ``perspective'' in this context.

Thank you for pointing this out. We meant to say that there are multiple ways to extend our model to account for more biological relevant behaviors. We replaced the word “perspective’’ by “aspect” in the manuscript. We would be happy to change the wording should it still be confusing to the reviewer.

6. The legibility of the figures could be improved by making them colorblind-friendly and making the error bars more distinct.

Thank you for your suggestion and we appreciate the reviewer’s concern. Unfortunately, we did not find any specific guidelines for PLoS Computational Biology regarding these aspects. However, we will be happy to upgrade the figures according to any editors’ suggestions at the production stage, should it be requested.

Response to Reviewer 2

In this well written piece, the authors pursue their line of research on the introduction of replica methods to describe the dynamics of finite (and potentially small) random neural networks. This research is certainly interesting and opens the way to new discoveries, some potentially interesting for biology. In this paper, the authors extend their methodology (both theoretically and numerically) to address the question of the characterization of metastable states in networks that include excitation, inhibition and exponential firing rates. The originality and main contribution of the paper lies in a clever use of the "resolvent formalism" instead of reduction to a delay equation that was used in previous works; this method for solving functional equations that involves infinite series that may be divergent, a problem clearly discussed in the manuscript. They combine this method to the use singular perturbation theory. This allows the authors to identify and describe pseudo-equilibria. Ample numerical simulations of the system are provided. They are performed with care, using advanced numerical methods based on Padé approximants that also appropriately handle possible divergences in Taylor series by considering rational fractions instead of polynomials, and that largely expand the range of convergence of numerical approximation.

The authors use these techniques to compute the nonlinear transfer function and their moments, with a particular focus on finite-size effects. They next look for fixed points of the equation and multi-stability, together with switches between equilibria in an E/I network, with a quite fine analysis of a (probably pitchfork or saddle-node) bifurcation of the system in response to changes in the excitation strength. The discussion is well thought and rich, and covers both questions related to the biological relevance of the findings, as well as further technical considerations that are deep and interesting. All derivations are provided in a supplementary file that is brief and clear. All derivations were checked and seem correct.

While the Referee likes this paper, it seems that this paper belongs more to the domain of stochastic analysis and theory rather than addresses a specifically formulated question in biology. The link with biology is well presented, but it did not appear to the Referee that this link was strong enough to warrant publication in Plos CB, whose mandate is to publish articles that "should model aspects of biological systems, demonstrate both methodological and scientific novelty, and provide profound new biological insights". Here I find that while methodological and scientific novelty, as well as rigor, are present, we are missing a clear and strong connection to biology both in the motivation and in the insight it provides.

We thank the reviewer for the favorable comments about our manuscript. The reviewer's main concern about biological relevance was also raised by Reviewer 1. Here, we address this concern in similar words.

We apologize for not clearly stating our biological motivation. To address this point, we rewrote the introduction of the manuscript to showcase the key motivation for our approach. This motivation is to account for the role that individual inputs can play in shaping the spiking output of a neuron. This is by contrast with most commonly used computational frameworks, which either ignore such individual impacts (e.g., classical mean-field models) or resist any analytical treatments. For these models, it is unclear, or at the very least very computationally expensive, to estimate the parametric dependences of the neural activity on the key parameters specifying neural circuits. Such parameters include the typical finite number of neuronal inputs, as well as the sizes of these inputs.

Here, we would like to complement the points made in the introduction by arguing that being able to relate neural variability and network parameters quantitatively is of great biological significance. We hope to achieve this by discussing experimental results from the Priebe and Seidemann labs at UT Austin, which we also mention in response to Reviewer 2. We do not include these results in the main manuscript as they will form the basis of a forthcoming paper by the Priebe and Seidemann labs. These results are presented in Fig. R1. A, where one can see that the mean and the variance of the membrane voltage of V1 neurons increase with stimulus onset in awake behaving monkeys. More generally, measurement in the Priebe and Seidemann labs shows that the mean voltage and the voltage variability jointly increase with the stimulus drive. This is contrary to similar measurements in other mammals, where variability quenching has been reported in response to stimulus presentation.

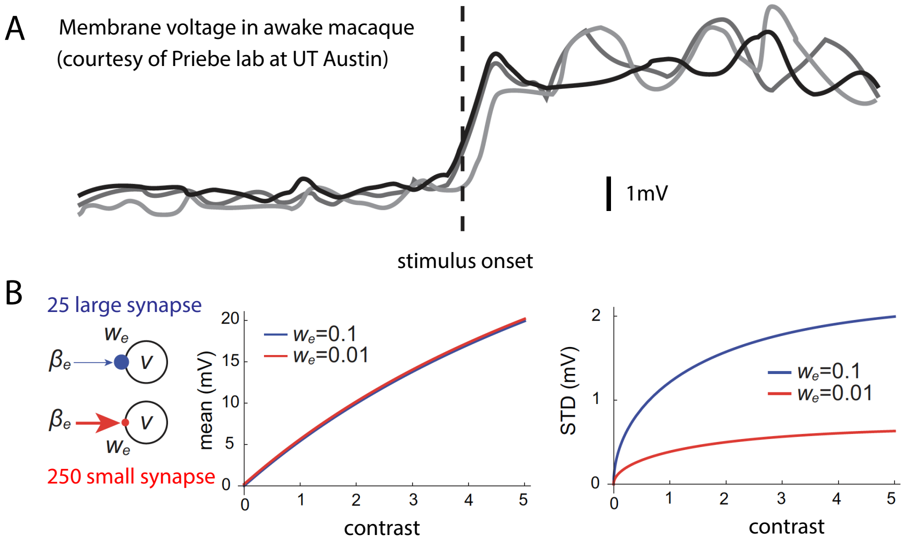

Fig. R1. **Measurement of membrane voltage in awake macaque.** Panel A: Sample measurements of the voltage time series around the stimulus onset. (Data from private communication with Priebe lab at UT Austin.) Panel B: The RMF method can capture the correct behavior of increasing variability.

We claim that a joint increase of the mean voltage and of the voltage variability cannot be explained within the confines of classical mean-field approaches. Indeed, classical thermodynamic limits ($1/N$ scaling) can only capture the proper mean response at the cost of a vanishing variability due to usual population averaging. Balanced limits ($1/\sqrt{N}$ scaling) can reproduce the proper variability dependence on the stimulus contrast but only at the cost of producing a nearly constant mean response. We believe both these mean-field approaches fails to appropriately capture the neural response because they neglect finite-size effects. To remedy this point, one can apply the RMF framework to a realistic conductance-based neural network. Such treatment shows that all aspect of the measured neuronal response can be recovered if the drive is primarily mediated by a few large synapses (< 50) with effective weights falling within a biologically plausible range ($w < 0.1$ms) (see Fig. R1. B). By biologically feasible, we mean that a single spiking event does not lead to larger than 0.5 mV fluctuations in the membrane voltages, as verified experimentally. This simple analysis argues that finite-size effects alone can account for the joint variation of the voltage mean and of the voltage variability.

The question addressed above is simple enough to be addressed in the RMF limit of a biophysically detailed model, such as a conductance-based one. When the questions at stake bare on collective aspects of the neural dynamics, such as in metastable dynamics, we expect the detailed structure of the network to play the central role. In the context of metastable dynamics, we believe that our proposed EGL models exhibit the satisfactory level of biologically relevance. In this regard, note that EGL neurons can produce the appropriate scaling of mean and variability, as opposed to other mean-field approaches. More generally, we would also like to point out that our analysis can be carried out for arbitrary topology and does not rely on any form of quenched-disorder hypothesis. We believe this to be quite a unique feature of our approach.

Descriptions of the Changes in the Manuscript

We have made substantial changes to the manuscript while preserving the overall structure. Here, we give a full list of the changes, excluding the corrections of grammatical errors or modifications of wording, which can be easily noted in the difference-highlighted version. In the following list, the line number is within the difference-highlighted version.

Introduction

- **[Line No. 3 - 33]** In the first paragraph, we added more details about the biological experimental evidence explaining the origination and importance of neural variability, which provided us the motivation of our modeling.
- **[Line No. 41 - 93]** In the second paragraph, we removed the modeling details and benefits for later discussion (L.N. 105 - 112). We added comparisons between the TMF and RMF limits in this paragraph to clarify the limitations of existing classical mean-field approaches.
- **[Line No. 134 - 149]** In the fourth paragraph, we elaborated on the capability of our models.
- **[Line No. 159 - 174]** In the fifth paragraph, we stressed the novelty and originality of our models and methods by providing two conceptual insights.

Modeling and theory

- **[Line No. 188 - 307]** We added two sections "Replica-mean-field framework for spiking networks" and "Mean-field limits for a simple intensity-based spiking network" aiming to provide a more pedagogical description to the RMF methods.
- **[Line No. 324 - 341]** We wrote down the stochastic intensity dynamics of our EGL models in the analogical form of the linear Galves-Löcherbach models and discussed the similarities and differences.
- **[Line No. 365 - 373]** This part was modified and moved to the introduction (L.N. 65 - 80).
- **[Line No. 457 - 464]** We analyzed the delay differential equation and provide motivations for our singular perturbative methods.
- **[Line No. 509 - 512]** We elaborated on the evidence supporting our claim, as requested by the reviewers.

Numerical methods

- **[Line No. 540 - 544]** We modified the statement of our numerical methods to make it clearer.
- **[Line No. 569 - 371]** We removed the obsolete statement.
- **[Line No. 574 - 597]** We restructured the introduction to the Padé approximants method and provided more intuition to make it easier to follow.
- **[Line No. 623 - 655]** We added the section "Computational efficiency" to demonstrate the supremacy of the RMF methods, as requested by the reviewers.

Results

- **[Line No. 931 - 948]** We added a paragraph with the analyze of the correlation structure of our bistable networks to support our claim, as requested by the reviewers.
- **[Line No. 1165 - 1191]** We modified and elaborated the paragraph describing the prediction of transition times.

Figures

- **[Fig. 1]** We added example dynamics of the original network to panel (a). We also added the citation of this figure in the caption.
- **[Fig. 4]** We added this figure for the section "Computational efficiency".
- **[Fig. 5]** We changed the color scheme in panel (c). Also, we added more explanation to the computing methods of the values and error bars.
- **[Fig. 6 - 11]** We added more explanation to the computing methods of the values and error bars.
- **[Fig. 12]** We added this figure for the text of analyzing the correlation structure (L.N. 941 - 959).
- **[Fig. 14]** We added this figure for the text of prediction of transition times (L.N. 1176 - 1203).
