# Supplementary material for: Metastable spiking networks in the replica-mean-field limit
Source: PLoS Comput Biol. 2022 Jun 17;18(6):e1010215. doi: 10.1371/journal.pcbi.1010215 (PMC9246178; doi:10.1371/journal.pcbi.1010215)
Supplement: Attachment — Submitted filename: Response Letter and Description of Changes_Second Revision.pdf [file pcbi.1010215.s004.pdf]

Revision of Manuscript PCOMPBIOL-D-21-01383

Luyan Yu and Thibaud Taillefumier

Dear Editors,

Thank you for taking the time to review our manuscript titled “Metastable networks in the replica-mean-field limit”. This letter is a response to the reviewers’ comments and a description of the changes to the manuscript. We appreciate your support.

Sincerely
Luyan Yu
Thibaud Taillefumier

Response to Editor’s Comments

1. You really do need to fix the issue regarding Galves-Locherbach references mentioned in the reviewer comments below. As the reviewer notes, in the computational neuroscience, this model is referred to as a linear non-linear Poisson (LNP) model with canonical reference to Pillow et. al. 2000 or a spike response model with canonical reference to Gerstner's book. The simple fix is to use LNP throughout the manuscript and restrict a Galves-Locherbach reference to a single line in the introduction pointing out that some circles refer to it as Galves-Locherbach model.

We appreciate the editor and the reviewer for pointing out this reference problem. We added the references for the LNP models and the relationships with our models in the section “Finite-size linear-exponential reset neuronal dynamics” under “Modeling and theory” (L.N. 259 – 262). Also, we refer to our model as the linear-exponential reset (LER) model instead of the exponential Galves-Locherbach (EGL) model.

2. Regarding biological significance, I very much appreciated the preview of your forthcoming work applying this technique and your reasons for withholding those results at this time. However, I would urge you to expand your discussion by a single paragraph simply outlining how this work could be applied without going into the details that you submitted in response to review.

We appreciate this suggestion and added a paragraph in section “Biophysical relevance and limitations” under “Discussion”. This paragraph gives an outline for the application of our method (L.N. 958 – 967).

Descriptions of the Changes in the Manuscript

In this revision, changes are made mainly to account for the two aspects from the editor’s comments. Here, we give a complete list of the changes, excluding the corrections of grammatical errors or wording modifications, which can be easily noted in the difference-highlighted version. In the following list, the line number is within the difference-highlighted version.

Introduction

- **[Line No. 68]** We changed the name of our model from the exponential Galves-Löcherbach (EGL) model to the linear-exponential reset (LER) model. Also, we change all the abbreviations from EGL to LER in all occurrences.

Modeling and theory

- **[Line No. 262 - 267]** We added the references to the linear-nonlinear Poisson (LNP) models and the relationships with our models.
- **[Line No. 294 - 295]** We removed the reference to the Galves-Löcherbach model.
- **[Line No. 315]** We removed the reference to the Galves-Löcherbach model.
- **[Line No. 381 - 383]** We removed the reference to the Galves-Löcherbach model.

Discussion

- **[Line No. 969 - 978]** We added a paragraph outlining the application of our method.

Acknowledgment

- **[Line No. 1122 - 1126]** We removed the funding information according to the PLOS guidelines.
